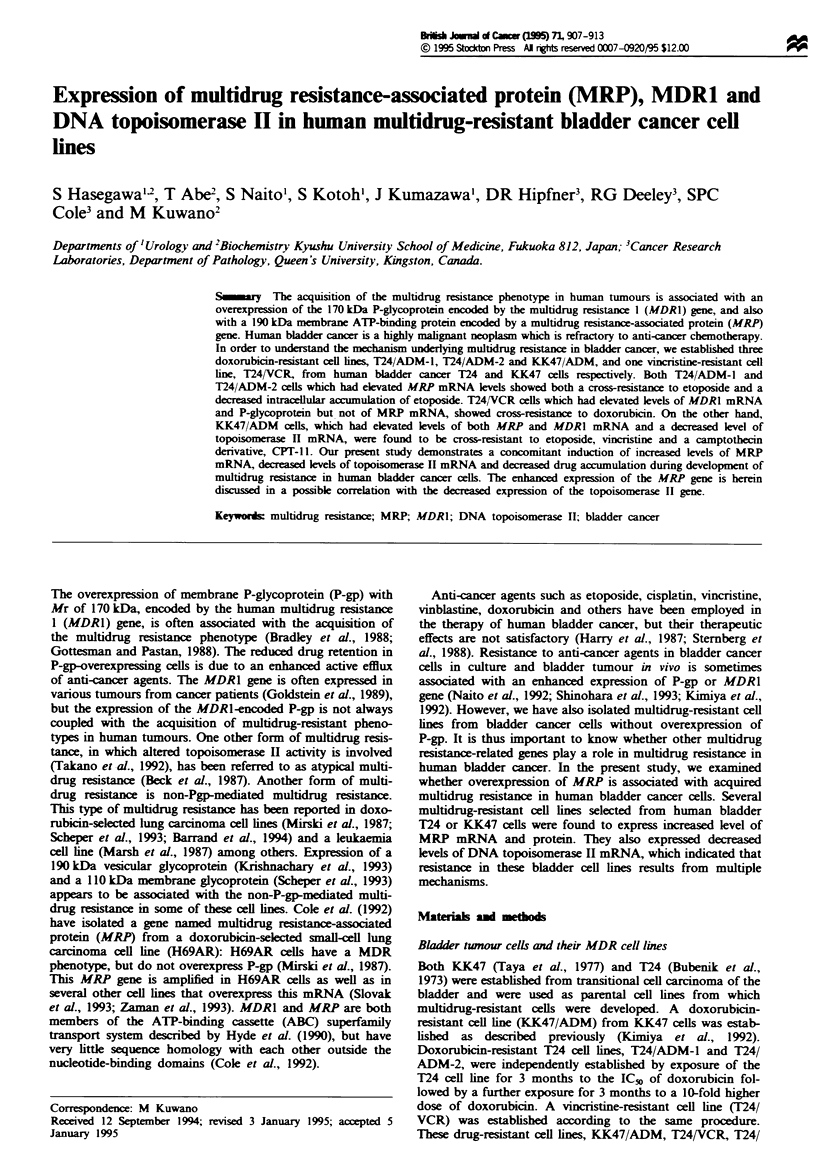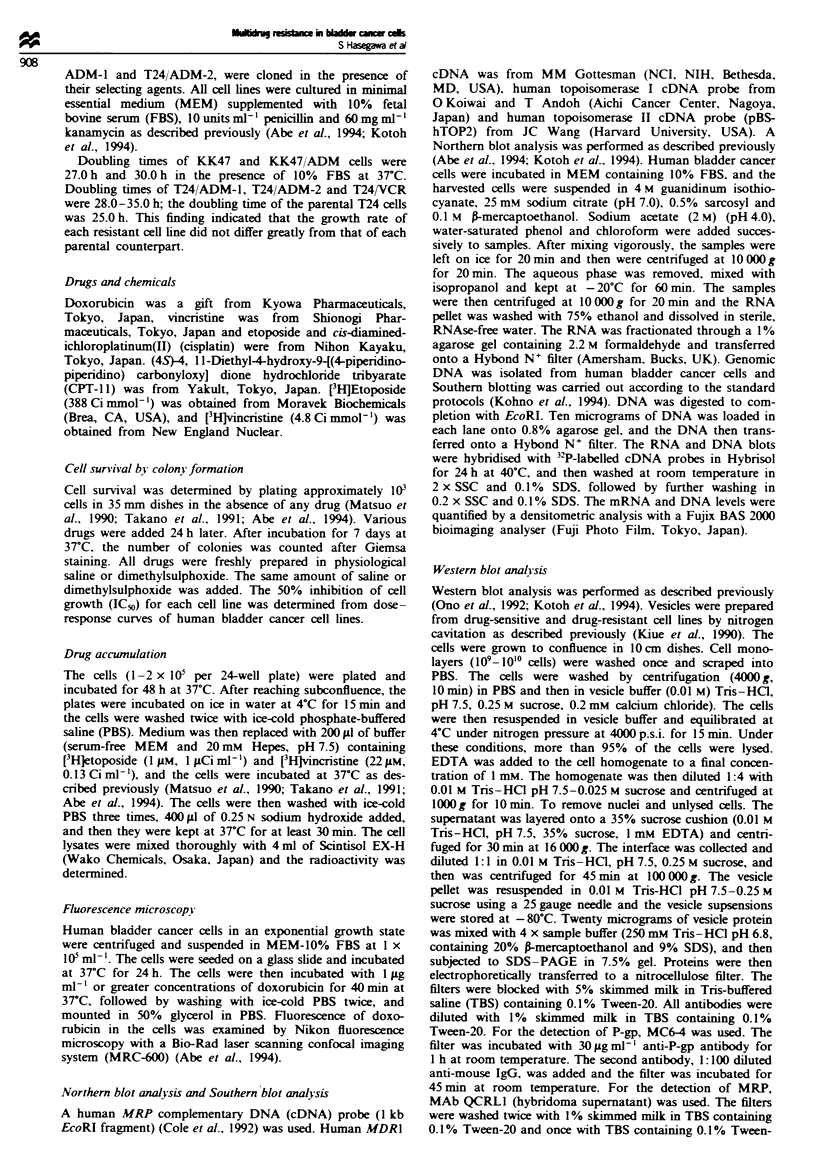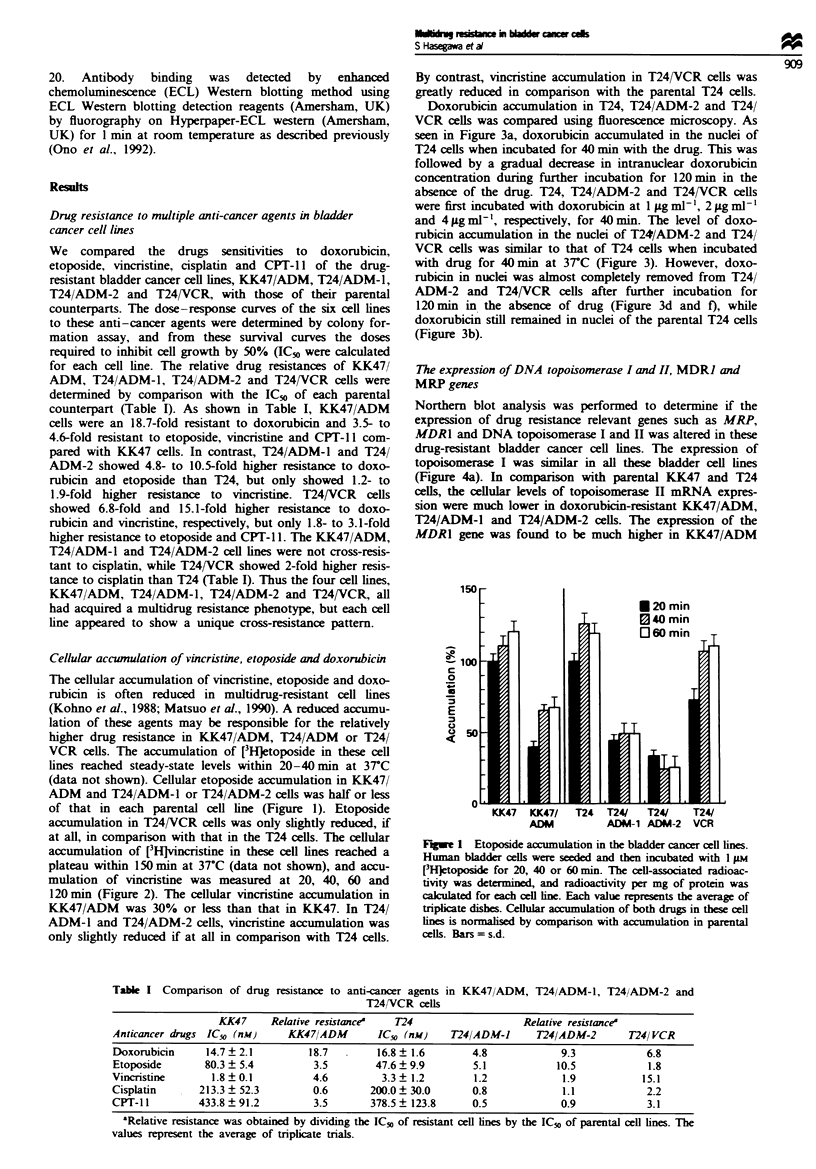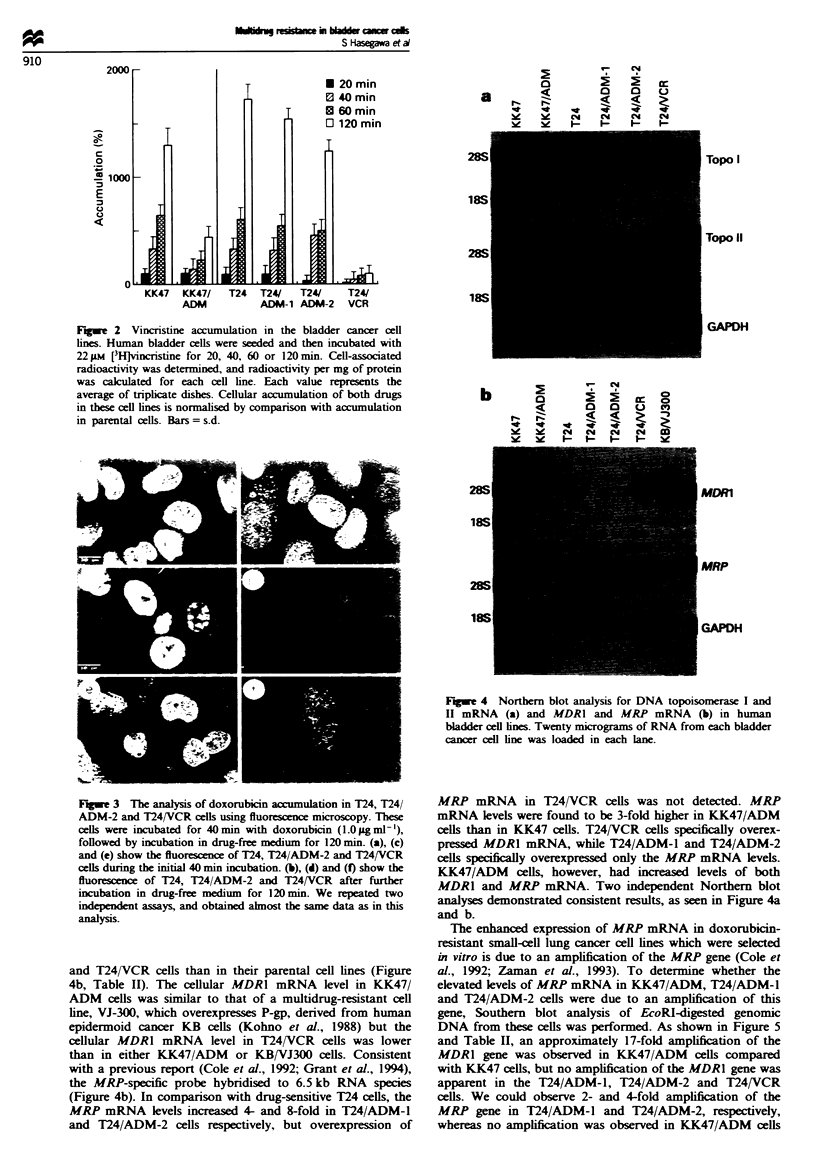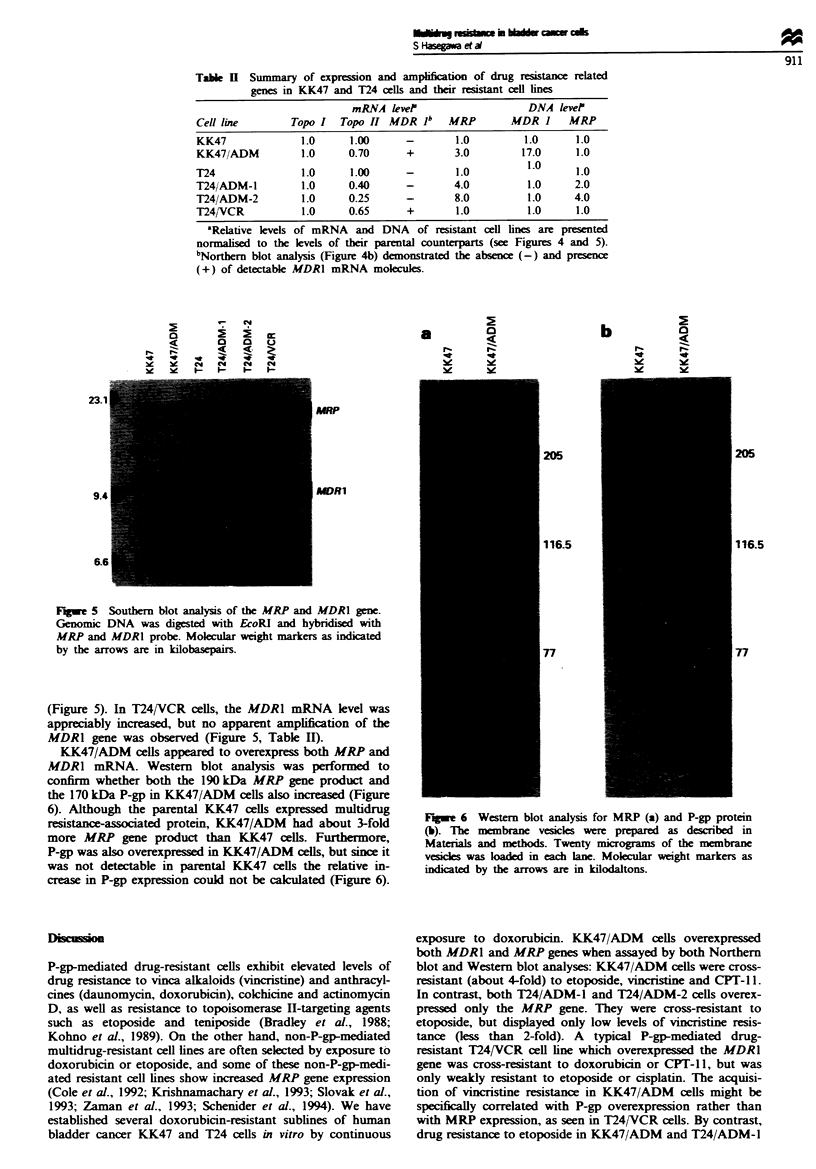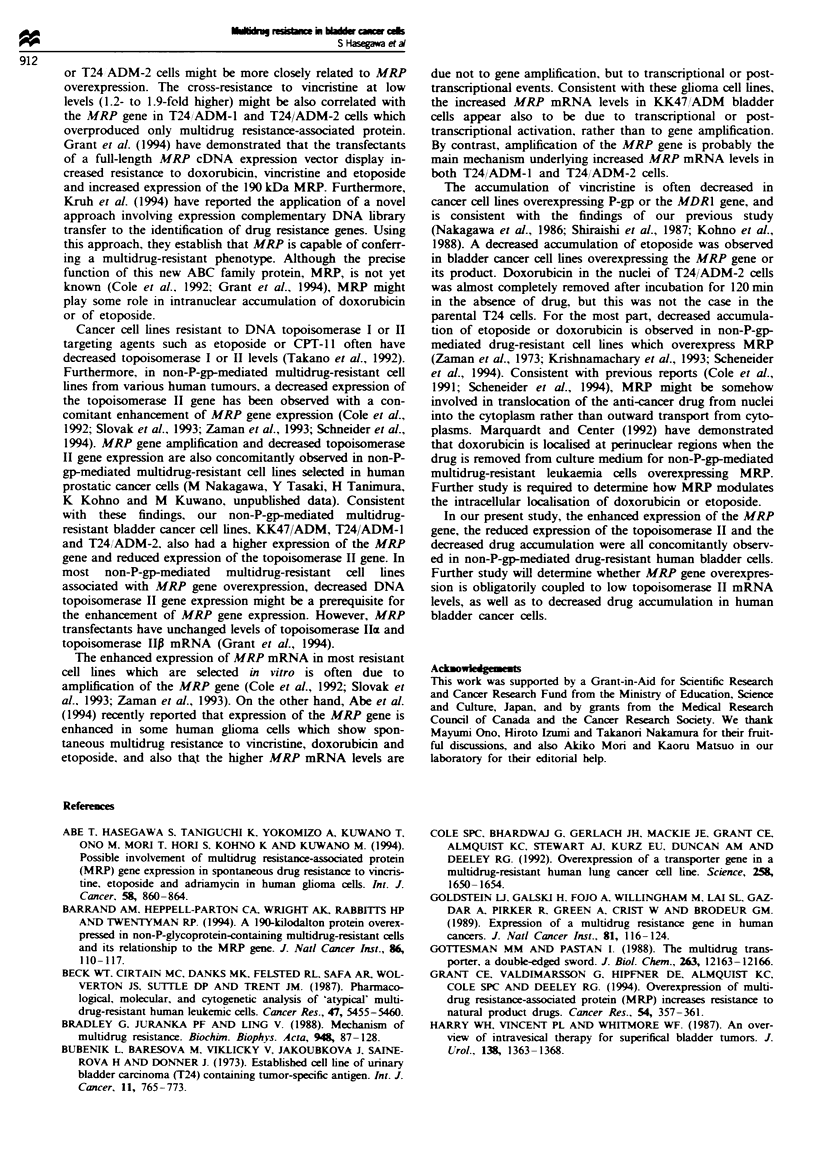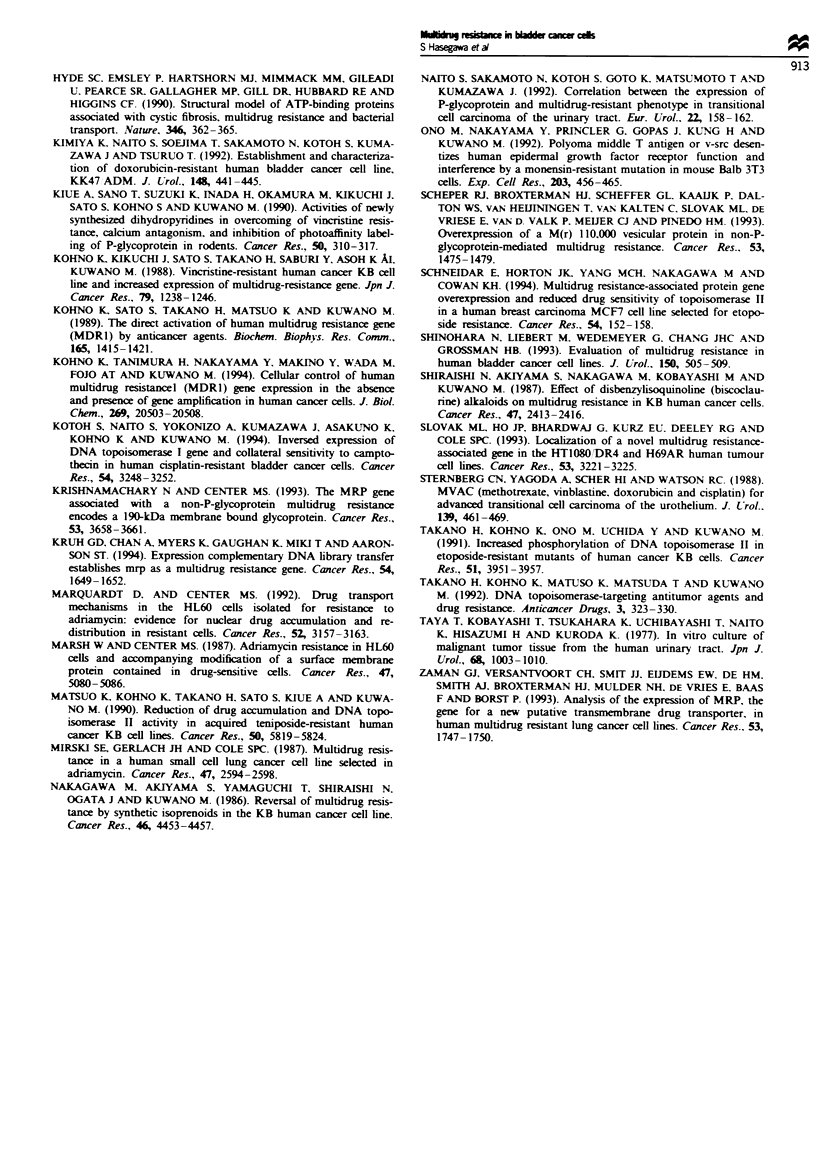# Expression of multidrug resistance-associated protein (MRP), MDR1 and DNA topoisomerase II in human multidrug-resistant bladder cancer cell lines.

**DOI:** 10.1038/bjc.1995.177

**Published:** 1995-05

**Authors:** S. Hasegawa, T. Abe, S. Naito, S. Kotoh, J. Kumazawa, D. R. Hipfner, R. G. Deeley, S. P. Cole, M. Kuwano

**Affiliations:** Department of Urology, Kyushu University School of Medicine, Fukuoka, Japan.

## Abstract

**Images:**


© 1995 Stockton Press All rights reserved 0007-0920/95 $12.00

### Expression of multidrug resistance-associated protein (MRP), MDR1 and DNA topoisomerase II in human multidrug-resistant bladder cancer cell lines

S Hasegawa[1,2], T Abe[2], S Naito[1], S Kotoh[1], J Kumazawa[1], DR Hipfner[3], RG Deeley[3], SPC Cole[3] and M Kuwano[2]

*Departments of [1]Urology and [2]Biochemistry Kyushu University School of Medicine, Fukuoka 812, Japan; [3]Cancer Research Laboratories, Department of Pathology, Queen's University, Kingston, Canada.*

**Summary** The acquisition of the multidrug resistance phenotype in human tumours is associated with an overexpression of the 170 kDa P-glycoprotein encoded by the multidrug resistance 1 (*MDR*1) gene, and also with a 190 kDa membrane ATP-binding protein encoded by a multidrug resistance-associated protein (*MRP*) gene. Human bladder cancer is a highly malignant neoplasm which is refractory to anti-cancer chemotherapy. In order to understand the mechanism underlying multidrug resistance in bladder cancer, we established three doxorubicin-resistant cell lines, T24/ADM-1, T24/ADM-2 and KK47/ADM, and one vincristine-resistant cell line, T24/VCR, from human bladder cancer T24 and KK47 cells respectively. Both T24/ADM-1 and T24/ADM-2 cells which had elevated *MRP* mRNA levels showed both a cross-resistance to etoposide and a decreased intracellular accumulation of etoposide. T24/VCR cells which had elevated levels of *MDR*1 mRNA and P-glycoprotein but not of MRP mRNA, showed cross-resistance to doxorubicin. On the other hand, KK47/ADM cells, which had elevated levels of both *MRP* and *MDR*1 mRNA and a decreased level of topoisomerase II mRNA, were found to be cross-resistant to etoposide, vincristine and a camptothecin derivative, CPT-11. Our present study demonstrates a concomitant induction of increased levels of MRP mRNA, decreased levels of topoisomerase II mRNA and decreased drug accumulation during development of multidrug resistance in human bladder cancer cells. The enhanced expression of the *MRP* gene is herein discussed in a possible correlation with the decreased expression of the topoisomerase II gene.

**Keywords:** multidrug resistance; MRP; *MDR*1; DNA topoisomerase II; bladder cancer

The overexpression of membrane P-glycoprotein (P-gp) with *M*r of 170 kDa, encoded by the human multidrug resistance 1 (*MDR*1) gene, is often associated with the acquisition of the multidrug resistance phenotype (Bradley *et al.*, 1988; Gottesman and Pastan, 1988). The reduced drug retention in P-gp-overexpressing cells is due to an enhanced active efflux of anti-cancer agents. The *MDR*1 gene is often expressed in various tumours from cancer patients (Goldstein *et al.*, 1989), but the expression of the *MDR*1-encoded P-gp is not always coupled with the acquisition of multidrug-resistant phenotypes in human tumours. One other form of multidrug resistance, in which altered topoisomerase II activity is involved (Takano *et al.*, 1992), has been referred to as atypical multidrug resistance (Beck *et al.*, 1987). Another form of multidrug resistance is non-Pgp-mediated multidrug resistance. This type of multidrug resistance has been reported in doxorubicin-selected lung carcinoma cell lines (Mirski *et al.*, 1987; Scheper *et al.*, 1993; Barrand *et al.*, 1994) and a leukaemia cell line (Marsh *et al.*, 1987) among others. Expression of a 190 kDa vesicular glycoprotein (Krishnachary *et al.*, 1993) and a 110 kDa membrane glycoprotein (Scheper *et al.*, 1993) appears to be associated with the non-P-gp-mediated multidrug resistance in some of these cell lines. Cole *et al.* (1992) have isolated a gene named multidrug resistance-associated protein (*MRP*) from a doxorubicin-selected small-cell lung carcinoma cell line (H69AR): H69AR cells have a MDR phenotype, but do not overexpress P-gp (Mirski *et al.*, 1987). This *MRP* gene is amplified in H69AR cells as well as in several other cell lines that overexpress this mRNA (Slovak *et al.*, 1993; Zaman *et al.*, 1993). *MDR*1 and *MRP* are both members of the ATP-binding cassette (ABC) superfamily transport system described by Hyde *et al.* (1990), but have very little sequence homology with each other outside the nucleotide-binding domains (Cole *et al.*, 1992).

Correspondence: M Kuwano

Received 12 September 1994; revised 3 January 1995; accepted 5 January 1995

Anti-cancer agents such as etoposide, cisplatin, vincristine, vinblastine, doxorubicin and others have been employed in the therapy of human bladder cancer, but their therapeutic effects are not satisfactory (Harry *et al.*, 1987; Sternberg *et al.*, 1988). Resistance to anti-cancer agents in bladder cancer cells in culture and bladder tumour *in vivo* is sometimes associated with an enhanced expression of P-gp or *MDR*1 gene (Naito *et al.*, 1992; Shinohara *et al.*, 1993; Kimiya *et al.*, 1992). However, we have also isolated multidrug-resistant cell lines from bladder cancer cells without overexpression of P-gp. It is thus important to know whether other multidrug resistance-related genes play a role in multidrug resistance in human bladder cancer. In the present study, we examined whether overexpression of *MRP* is associated with acquired multidrug resistance in human bladder cancer cells. Several multidrug-resistant cell lines selected from human bladder T24 or KK47 cells were found to express increased level of MRP mRNA and protein. They also expressed decreased levels of DNA topoisomerase II mRNA, which indicated that resistance in these bladder cell lines results from multiple mechanisms.

### Materials and methods

### *Bladder tumour cells and their MDR cell lines*

Both KK47 (Taya *et al.*, 1977) and T24 (Bubenik *et al.*, 1973) were established from transitional cell carcinoma of the bladder and were used as parental cell lines from which multidrug-resistant cells were developed. A doxorubicin-resistant cell line (KK47/ADM) from KK47 cells was established as described previously (Kimiya *et al.*, 1992). Doxorubicin-resistant T24 cell lines, T24/ADM-1 and T24/ADM-2, were independently established by exposure of the T24 cell line for 3 months to the $IC_{50}$ of doxorubicin followed by a further exposure for 3 months to a 10-fold higher dose of doxorubicin. A vincristine-resistant cell line (T24/VCR) was established according to the same procedure. These drug-resistant cell lines, KK47/ADM, T24/VCR, T24/

ADM-1 and T24/ADM-2, were cloned in the presence of their selecting agents. All cell lines were cultured in minimal essential medium (MEM) supplemented with 10% fetal bovine serum (FBS), 10 units $ml^{-1}$ penicillin and 60 mg $ml^{-1}$ kanamycin as described previously (Abe *et al.*, 1994; Kotoh *et al.*, 1994).

Doubling times of KK47 and KK47/ADM cells were 27.0 h and 30.0 h in the presence of 10% FBS at 37°C. Doubling times of T24/ADM-1, T24/ADM-2 and T24/VCR were 28.0–35.0 h; the doubling time of the parental T24 cells was 25.0 h. This finding indicated that the growth rate of each resistant cell line did not differ greatly from that of each parental counterpart.

### *Drugs and chemicals*

Doxorubicin was a gift from Kyowa Pharmaceuticals, Tokyo, Japan, vincristine was from Shionogi Pharmaceuticals, Tokyo, Japan and etoposide and *cis*-diaminedichloroplatinum(II) (cisplatin) were from Nihon Kayaku, Tokyo, Japan. (4*S*)-4, 11-Diethyl-4-hydroxy-9-[(4-piperidino-piperidino) carbonyloxy] dione hydrochloride tribyarate (CPT-11) was from Yakult, Tokyo, Japan. [$^3$H]Etoposide (388 Ci $mmol^{-1}$) was obtained from Moravek Biochemicals (Brea, CA, USA), and [$^3$H]vincristine (4.8 Ci $mmol^{-1}$) was obtained from New England Nuclear.

### *Cell survival by colony formation*

Cell survival was determined by plating approximately $10^3$ cells in 35 mm dishes in the absence of any drug (Matsuo *et al.*, 1990; Takano *et al.*, 1991; Abe *et al.*, 1994). Various drugs were added 24 h later. After incubation for 7 days at 37°C, the number of colonies was counted after Giemsa staining. All drugs were freshly prepared in physiological saline or dimethylsulphoxide. The same amount of saline or dimethylsulphoxide was added. The 50% inhibition of cell growth ($IC_{50}$) for each cell line was determined from dose–response curves of human bladder cancer cell lines.

### *Drug accumulation*

The cells ($1-2 \times 10^5$ per 24-well plate) were plated and incubated for 48 h at 37°C. After reaching subconfluence, the plates were incubated on ice in water at 4°C for 15 min and the cells were washed twice with ice-cold phosphate-buffered saline (PBS). Medium was then replaced with 200 µl of buffer (serum-free MEM and 20 mM Hepes, pH 7.5) containing [$^3$H]etoposide (1 µM, 1 µCi $ml^{-1}$) and [$^3$H]vincristine (22 µM, 0.13 Ci $ml^{-1}$), and the cells were incubated at 37°C as described previously (Matsuo *et al.*, 1990; Takano *et al.*, 1991; Abe *et al.*, 1994). The cells were then washed with ice-cold PBS three times, 400 µl of 0.25 N sodium hydroxide added, and then they were kept at 37°C for at least 30 min. The cell lysates were mixed thoroughly with 4 ml of Scintisol EX-H (Wako Chemicals, Osaka, Japan) and the radioactivity was determined.

### *Fluorescence microscopy*

Human bladder cancer cells in an exponential growth state were centrifuged and suspended in MEM-10% FBS at $1 \times 10^5$ $ml^{-1}$. The cells were seeded on a glass slide and incubated at 37°C for 24 h. The cells were then incubated with 1 µg $ml^{-1}$ or greater concentrations of doxorubicin for 40 min at 37°C, followed by washing with ice-cold PBS twice, and mounted in 50% glycerol in PBS. Fluorescence of doxorubicin in the cells was examined by Nikon fluorescence microscopy with a Bio-Rad laser scanning confocal imaging system (MRC-600) (Abe *et al.*, 1994).

### *Northern blot analysis and Southern blot analysis*

A human *MRP* complementary DNA (cDNA) probe (1 kb *Eco*RI fragment) (Cole *et al.*, 1992) was used. Human *MDR*1 cDNA was from MM Gottesman (NCI, NIH, Bethesda, MD, USA), human topoisomerase I cDNA probe from O Koiwai and T Andoh (Aichi Cancer Center, Nagoya, Japan) and human topoisomerase II cDNA probe (pBS-hTOP2) from JC Wang (Harvard University, USA). A Northern blot analysis was performed as described previously (Abe *et al.*, 1994; Kotoh *et al.*, 1994). Human bladder cancer cells were incubated in MEM containing 10% FBS, and the harvested cells were suspended in 4 M guanidinum isothiocyanate, 25 mM sodium citrate (pH 7.0), 0.5% sarcosyl and 0.1 M β-mercaptoethanol. Sodium acetate (2 M) (pH 4.0), water-saturated phenol and chloroform were added successively to samples. After mixing vigorously, the samples were left on ice for 20 min and then were centrifuged at 10 000 *g* for 20 min. The aqueous phase was removed, mixed with isopropanol and kept at −20°C for 60 min. The samples were then centrifuged at 10 000 *g* for 20 min and the RNA pellet was washed with 75% ethanol and dissolved in sterile, RNAse-free water. The RNA was fractionated through a 1% agarose gel containing 2.2 M formaldehyde and transferred onto a Hybond $N^+$ filter (Amersham, Bucks, UK). Genomic DNA was isolated from human bladder cancer cells and Southern blotting was carried out according to the standard protocols (Kohno *et al.*, 1994). DNA was digested to completion with *Eco*RI. Ten micrograms of DNA was loaded in each lane onto 0.8% agarose gel, and the DNA then transferred onto a Hybond $N^+$ filter. The RNA and DNA blots were hybridised with $^{32}$P-labelled cDNA probes in Hybrisol for 24 h at 40°C, and then washed at room temperature in 2 × SSC and 0.1% SDS, followed by further washing in 0.2 × SSC and 0.1% SDS. The mRNA and DNA levels were quantified by a densitometric analysis with a Fujix BAS 2000 bioimaging analyser (Fuji Photo Film, Tokyo, Japan).

### *Western blot analysis*

Western blot analysis was performed as described previously (Ono *et al.*, 1992; Kotoh *et al.*, 1994). Vesicles were prepared from drug-sensitive and drug-resistant cell lines by nitrogen cavitation as described previously (Kiue *et al.*, 1990). The cells were grown to confluence in 10 cm dishes. Cell monolayers ($10^9-10^{10}$ cells) were washed once and scraped into PBS. The cells were washed by centrifugation (4000 *g*, 10 min) in PBS and then in vesicle buffer (0.01 M) Tris–HCl, pH 7.5, 0.25 M sucrose, 0.2 mM calcium chloride). The cells were then resuspended in vesicle buffer and equilibrated at 4°C under nitrogen pressure at 4000 p.s.i. for 15 min. Under these conditions, more than 95% of the cells were lysed. EDTA was added to the cell homogenate to a final concentration of 1 mM. The homogenate was then diluted 1:4 with 0.01 M Tris–HCl pH 7.5–0.025 M sucrose and centrifuged at 1000 *g* for 10 min. To remove nuclei and unlysed cells. The supernatant was layered onto a 35% sucrose cushion (0.01 M Tris–HCl, pH 7.5, 35% sucrose, 1 mM EDTA) and centrifuged for 30 min at 16 000 *g*. The interface was collected and diluted 1:1 in 0.01 M Tris–HCl, pH 7.5, 0.25 M sucrose, and then was centrifuged for 45 min at 100 000 *g*. The vesicle pellet was resuspended in 0.01 M Tris-HCl pH 7.5–0.25 M sucrose using a 25 gauge needle and the vesicle supsensions were stored at −80°C. Twenty micrograms of vesicle protein was mixed with 4 × sample buffer (250 mM Tris–HCl pH 6.8, containing 20% β-mercaptoethanol and 9% SDS), and then subjected to SDS–PAGE in 7.5% gel. Proteins were then electrophoretically transferred to a nitrocellulose filter. The filters were blocked with 5% skimmed milk in Tris-buffered saline (TBS) containing 0.1% Tween-20. All antibodies were diluted with 1% skimmed milk in TBS containing 0.1% Tween-20. For the detection of P-gp, MC6-4 was used. The filter was incubated with 30 µg $ml^{-1}$ anti-P-gp antibody for 1 h at room temperature. The second antibody, 1:100 diluted anti-mouse IgG, was added and the filter was incubated for 45 min at room temperature. For the detection of MRP, MAb QCRL1 (hybridoma supernatant) was used. The filters were washed twice with 1% skimmed milk in TBS containing 0.1% Tween-20 and once with TBS containing 0.1% Tween-

20. Antibody binding was detected by enhanced chemoluminescence (ECL) Western blotting method using ECL Western blotting detection reagents (Amersham, UK) by fluorography on Hyperpaper-ECL western (Amersham, UK) for 1 min at room temperature as described previously (Ono *et al.*, 1992).

### Results

### *Drug resistance to multiple anti-cancer agents in bladder cancer cell lines*

We compared the drugs sensitivities to doxorubicin, etoposide, vincristine, cisplatin and CPT-11 of the drug-resistant bladder cancer cell lines, KK47/ADM, T24/ADM-1, T24/ADM-2 and T24/VCR, with those of their parental counterparts. The dose–response curves of the six cell lines to these anti-cancer agents were determined by colony formation assay, and from these survival curves the doses required to inhibit cell growth by 50% ($IC_{50}$ were calculated for each cell line. The relative drug resistances of KK47/ADM, T24/ADM-1, T24/ADM-2 and T24/VCR cells were determined by comparison with the $IC_{50}$ of each parental counterpart (Table I). As shown in Table I, KK47/ADM cells were an 18.7-fold resistant to doxorubicin and 3.5- to 4.6-fold resistant to etoposide, vincristine and CPT-11 compared with KK47 cells. In contrast, T24/ADM-1 and T24/ADM-2 showed 4.8- to 10.5-fold higher resistance to doxorubicin and etoposide than T24, but only showed 1.2- to 1.9-fold higher resistance to vincristine. T24/VCR cells showed 6.8-fold and 15.1-fold higher resistance to doxorubicin and vincristine, respectively, but only 1.8- to 3.1-fold higher resistance to etoposide and CPT-11. The KK47/ADM, T24/ADM-1 and T24/ADM-2 cell lines were not cross-resistant to cisplatin, while T24/VCR showed 2-fold higher resistance to cisplatin than T24 (Table I). Thus the four cell lines, KK47/ADM, T24/ADM-1, T24/ADM-2 and T24/VCR, all had acquired a multidrug resistance phenotype, but each cell line appeared to show a unique cross-resistance pattern.

### *Cellular accumulation of vincristine, etoposide and doxorubicin*

The cellular accumulation of vincristine, etoposide and doxorubicin is often reduced in multidrug-resistant cell lines (Kohno *et al.*, 1988; Matsuo *et al.*, 1990). A reduced accumulation of these agents may be responsible for the relatively higher drug resistance in KK47/ADM, T24/ADM or T24/VCR cells. The accumulation of [$^3$H]etoposide in these cell lines reached steady-state levels within 20–40 min at 37°C (data not shown). Cellular etoposide accumulation in KK47/ADM and T24/ADM-1 or T24/ADM-2 cells was half or less of that in each parental cell line (Figure 1). Etoposide accumulation in T24/VCR cells was only slightly reduced, if at all, in comparison with that in the T24 cells. The cellular accumulation of [$^3$H]vincristine in these cell lines reached a plateau within 150 min at 37°C (data not shown), and accumulation of vincristine was measured at 20, 40, 60 and 120 min (Figure 2). The cellular vincristine accumulation in KK47/ADM was 30% or less than that in KK47. In T24/ADM-1 and T24/ADM-2 cells, vincristine accumulation was only slightly reduced if at all in comparison with T24 cells. By contrast, vincristine accumulation in T24/VCR cells was greatly reduced in comparison with the parental T24 cells.

Doxorubicin accumulation in T24, T24/ADM-2 and T24/VCR cells was compared using fluorescence microscopy. As seen in Figure 3a, doxorubicin accumulated in the nuclei of T24 cells when incubated for 40 min with the drug. This was followed by a gradual decrease in intranuclear doxorubicin concentration during further incubation for 120 min in the absence of the drug. T24, T24/ADM-2 and T24/VCR cells were first incubated with doxorubicin at 1 μg ml$^{-1}$, 2 μg ml$^{-1}$ and 4 μg ml$^{-1}$, respectively, for 40 min. The level of doxorubicin accumulation in the nuclei of T24/ADM-2 and T24/VCR cells was similar to that of T24 cells when incubated with drug for 40 min at 37°C (Figure 3). However, doxorubicin in nuclei was almost completely removed from T24/ADM-2 and T24/VCR cells after further incubation for 120 min in the absence of drug (Figure 3d and f), while doxorubicin still remained in nuclei of the parental T24 cells (Figure 3b).

### *The expression of DNA topoisomerase I and II,* MDR*1 and* MRP *genes*

Northern blot analysis was performed to determine if the expression of drug resistance relevant genes such as *MRP*, *MDR*1 and DNA topoisomerase I and II was altered in these drug-resistant bladder cancer cell lines. The expression of topoisomerase I was similar in all these bladder cell lines (Figure 4a). In comparison with parental KK47 and T24 cells, the cellular levels of topoisomerase II mRNA expression were much lower in doxorubicin-resistant KK47/ADM, T24/ADM-1 and T24/ADM-2 cells. The expression of the *MDR*1 gene was found to be much higher in KK47/ADM

**Figure 1** Etoposide accumulation in the bladder cancer cell lines. Human bladder cells were seeded and then incubated with 1 μM [$^3$H]etoposide for 20, 40 or 60 min. The cell-associated radioactivity was determined, and radioactivity per mg of protein was calculated for each cell line. Each value represents the average of triplicate dishes. Cellular accumulation of both drugs in these cell lines is normalised by comparison with accumulation in parental cells. Bars = s.d.

**Table I** Comparison of drug resistance to anti-cancer agents in KK47/ADM, T24/ADM-1, T24/ADM-2 and T24/VCR cells

| Anticancer drugs | *KK47* *$IC_{50}$ (nM)* | *Relative resistance*[a] *KK47/ADM* | *T24* *$IC_{50}$ (nM)* | *Relative resistance*[a] *T24/ADM-1* | *T24/ADM-2* | *T24/VCR* |
|---|---|---|---|---|---|---|
| Doxorubicin | 14.7 ± 2.1 | 18.7 | 16.8 ± 1.6 | 4.8 | 9.3 | 6.8 |
| Etoposide | 80.3 ± 5.4 | 3.5 | 47.6 ± 9.9 | 5.1 | 10.5 | 1.8 |
| Vincristine | 1.8 ± 0.1 | 4.6 | 3.3 ± 1.2 | 1.2 | 1.9 | 15.1 |
| Cisplatin | 213.3 ± 52.3 | 0.6 | 200.0 ± 30.0 | 0.8 | 1.1 | 2.2 |
| CPT-11 | 433.8 ± 91.2 | 3.5 | 378.5 ± 123.8 | 0.5 | 0.9 | 3.1 |

[a]Relative resistance was obtained by dividing the $IC_{50}$ of resistant cell lines by the $IC_{50}$ of parental cell lines. The values represent the average of triplicate trials.

**Figure 2** Vincristine accumulation in the bladder cancer cell lines. Human bladder cells were seeded and then incubated with 22 μM [$^3$H]vincristine for 20, 40, 60 or 120 min. Cell-associated radioactivity was determined, and radioactivity per mg of protein was calculated for each cell line. Each value represents the average of triplicate dishes. Cellular accumulation of both drugs in these cell lines is normalised by comparison with accumulation in parental cells. Bars = s.d.

**Figure 3** The analysis of doxorubicin accumulation in T24, T24/ADM-2 and T24/VCR cells using fluorescence microscopy. These cells were incubated for 40 min with doxorubicin (1.0 μg ml$^{-1}$), followed by incubation in drug-free medium for 120 min. (**a**), (**c**) and (**e**) show the fluorescence of T24, T24/ADM-2 and T24/VCR cells during the initial 40 min incubation. (**b**), (**d**) and (**f**) show the fluorescence of T24, T24/ADM-2 and T24/VCR after further incubation in drug-free medium for 120 min. We repeated two independent assays, and obtained almost the same data as in this analysis.

and T24/VCR cells than in their parental cell lines (Figure 4b, Table II). The cellular *MDR*1 mRNA level in KK47/ADM cells was similar to that of a multidrug-resistant cell line, VJ-300, which overexpresses P-gp, derived from human epidermoid cancer KB cells (Kohno *et al.*, 1988) but the cellular *MDR*1 mRNA level in T24/VCR cells was lower than in either KK47/ADM or KB/VJ300 cells. Consistent with a previous report (Cole *et al.*, 1992; Grant *et al.*, 1994), the *MRP*-specific probe hybridised to 6.5 kb RNA species (Figure 4b). In comparison with drug-sensitive T24 cells, the *MRP* mRNA levels increased 4- and 8-fold in T24/ADM-1 and T24/ADM-2 cells respectively, but overexpression of

**Figure 4** Northern blot analysis for DNA topoisomerase I and II mRNA (**a**) and *MDR*1 and *MRP* mRNA (**b**) in human bladder cell lines. Twenty micrograms of RNA from each bladder cancer cell line was loaded in each lane.

*MRP* mRNA in T24/VCR cells was not detected. *MRP* mRNA levels were found to be 3-fold higher in KK47/ADM cells than in KK47 cells. T24/VCR cells specifically overexpressed *MDR*1 mRNA, while T24/ADM-1 and T24/ADM-2 cells specifically overexpressed only the *MRP* mRNA levels. KK47/ADM cells, however, had increased levels of both *MDR*1 and *MRP* mRNA. Two independent Northern blot analyses demonstrated consistent results, as seen in Figure 4a and b.

The enhanced expression of *MRP* mRNA in doxorubicin-resistant small-cell lung cancer cell lines which were selected *in vitro* is due to an amplification of the *MRP* gene (Cole *et al.*, 1992; Zaman *et al.*, 1993). To determine whether the elevated levels of *MRP* mRNA in KK47/ADM, T24/ADM-1 and T24/ADM-2 cells were due to an amplification of this gene, Southern blot analysis of *Eco*RI-digested genomic DNA from these cells was performed. As shown in Figure 5 and Table II, an approximately 17-fold amplification of the *MDR*1 gene was observed in KK47/ADM cells compared with KK47 cells, but no amplification of the *MDR*1 gene was apparent in the T24/ADM-1, T24/ADM-2 and T24/VCR cells. We could observe 2- and 4-fold amplification of the *MRP* gene in T24/ADM-1 and T24/ADM-2, respectively, whereas no amplification was observed in KK47/ADM cells

**Table II** Summary of expression and amplification of drug resistance related genes in KK47 and T24 cells and their resistant cell lines

| | mRNA level[a] | | | | DNA level[a] | |
|---|---|---|---|---|---|---|
| *Cell line* | *Topo I* | *Topo II* | *MDR 1*[b] | *MRP* | *MDR 1* | *MRP* |
| KK47 | 1.0 | 1.00 | – | 1.0 | 1.0 | 1.0 |
| KK47/ADM | 1.0 | 0.70 | + | 3.0 | 17.0 | 1.0 |
| T24 | 1.0 | 1.00 | – | 1.0 | 1.0 | 1.0 |
| T24/ADM-1 | 1.0 | 0.40 | – | 4.0 | 1.0 | 2.0 |
| T24/ADM-2 | 1.0 | 0.25 | – | 8.0 | 1.0 | 4.0 |
| T24/VCR | 1.0 | 0.65 | + | 1.0 | 1.0 | 1.0 |

[a]Relative levels of mRNA and DNA of resistant cell lines are presented normalised to the levels of their parental counterparts (see Figures 4 and 5). [b]Northern blot analysis (Figure 4b) demonstrated the absence (–) and presence (+) of detectable *MDR*1 mRNA molecules.

**Figure 5** Southern blot analysis of the *MRP* and *MDR*1 gene. Genomic DNA was digested with *Eco*RI and hybridised with *MRP* and *MDR*1 probe. Molecular weight markers as indicated by the arrows are in kilobasepairs.

**Figure 6** Western blot analysis for MRP (**a**) and P-gp protein (**b**). The membrane vesicles were prepared as described in Materials and methods. Twenty micrograms of the membrane vesicles was loaded in each lane. Molecular weight markers as indicated by the arrows are in kilodaltons.

(Figure 5). In T24/VCR cells, the *MDR*1 mRNA level was appreciably increased, but no apparent amplification of the *MDR*1 gene was observed (Figure 5, Table II).

KK47/ADM cells appeared to overexpress both *MRP* and *MDR*1 mRNA. Western blot analysis was performed to confirm whether both the 190 kDa *MRP* gene product and the 170 kDa P-gp in KK47/ADM cells also increased (Figure 6). Although the parental KK47 cells expressed multidrug resistance-associated protein, KK47/ADM had about 3-fold more *MRP* gene product than KK47 cells. Furthermore, P-gp was also overexpressed in KK47/ADM cells, but since it was not detectable in parental KK47 cells the relative increase in P-gp expression could not be calculated (Figure 6).

### Discussion

P-gp-mediated drug-resistant cells exhibit elevated levels of drug resistance to vinca alkaloids (vincristine) and anthracylcines (daunomycin, doxorubicin), colchicine and actinomycin D, as well as resistance to topoisomerase II-targeting agents such as etoposide and teniposide (Bradley *et al*., 1988; Kohno *et al*., 1989). On the other hand, non-P-gp-mediated multidrug-resistant cell lines are often selected by exposure to doxorubicin or etoposide, and some of these non-P-gp-mediated resistant cell lines show increased *MRP* gene expression (Cole *et al*., 1992; Krishnamachary *et al*., 1993; Slovak *et al*., 1993; Zaman *et al*., 1993; Schenider *et al*., 1994). We have established several doxorubicin-resistant sublines of human bladder cancer KK47 and T24 cells *in vitro* by continuous exposure to doxorubicin. KK47/ADM cells overexpressed both *MDR*1 and *MRP* genes when assayed by both Northern blot and Western blot analyses: KK47/ADM cells were cross-resistant (about 4-fold) to etoposide, vincristine and CPT-11. In contrast, both T24/ADM-1 and T24/ADM-2 cells overexpressed only the *MRP* gene. They were cross-resistant to etoposide, but displayed only low levels of vincristine resistance (less than 2-fold). A typical P-gp-mediated drug-resistant T24/VCR cell line which overexpressed the *MDR*1 gene was cross-resistant to doxorubicin or CPT-11, but was only weakly resistant to etoposide or cisplatin. The acquisition of vincristine resistance in KK47/ADM cells might be specifically correlated with P-gp overexpression rather than with MRP expression, as seen in T24/VCR cells. By contrast, drug resistance to etoposide in KK47/ADM and T24/ADM-1

or T24/ADM-2 cells might be more closely related to *MRP* overexpression. The cross-resistance to vincristine at low levels (1.2- to 1.9-fold higher) might be also correlated with the *MRP* gene in T24/ADM-1 and T24/ADM-2 cells which overproduced only multidrug resistance-associated protein. Grant *et al.* (1994) have demonstrated that the transfectants of a full-length *MRP* cDNA expression vector display increased resistance to doxorubicin, vincristine and etoposide and increased expression of the 190 kDa MRP. Furthermore, Kruh *et al.* (1994) have reported the application of a novel approach involving expression complementary DNA library transfer to the identification of drug resistance genes. Using this approach, they establish that *MRP* is capable of conferring a multidrug-resistant phenotype. Although the precise function of this new ABC family protein, MRP, is not yet known (Cole *et al.*, 1992; Grant *et al.*, 1994), MRP might play some role in intranuclear accumulation of doxorubicin or of etoposide.

Cancer cell lines resistant to DNA topoisomerase I or II targeting agents such as etoposide or CPT-11 often have decreased topoisomerase I or II levels (Takano *et al.*, 1992). Furthermore, in non-P-gp-mediated multidrug-resistant cell lines from various human tumours, a decreased expression of the topoisomerase II gene has been observed with a concomitant enhancement of *MRP* gene expression (Cole *et al.*, 1992; Slovak *et al.*, 1993; Zaman *et al.*, 1993; Schneider *et al.*, 1994). *MRP* gene amplification and decreased topoisomerase II gene expression are also concomitantly observed in non-P-gp-mediated multidrug-resistant cell lines selected in human prostatic cancer cells (M Nakagawa, Y Tasaki, H Tanimura, K Kohno and M Kuwano, unpublished data). Consistent with these findings, our non-P-gp-mediated multidrug-resistant bladder cancer cell lines, KK47/ADM, T24/ADM-1 and T24/ADM-2, also had a higher expression of the *MRP* gene and reduced expression of the topoisomerase II gene. In most non-P-gp-mediated multidrug-resistant cell lines associated with *MRP* gene overexpression, decreased DNA topoisomerase II gene expression might be a prerequisite for the enhancement of *MRP* gene expression. However, *MRP* transfectants have unchanged levels of topoisomerase II$\alpha$ and topoisomerase II$\beta$ mRNA (Grant *et al.*, 1994).

The enhanced expression of *MRP* mRNA in most resistant cell lines which are selected *in vitro* is often due to amplification of the *MRP* gene (Cole *et al.*, 1992; Slovak *et al.*, 1993; Zaman *et al.*, 1993). On the other hand, Abe *et al.* (1994) recently reported that expression of the *MRP* gene is enhanced in some human glioma cells which show spontaneous multidrug resistance to vincristine, doxorubicin and etoposide, and also that the higher *MRP* mRNA levels are due not to gene amplification, but to transcriptional or post-transcriptional events. Consistent with these glioma cell lines, the increased *MRP* mRNA levels in KK47/ADM bladder cells appear also to be due to transcriptional or post-transcriptional activation, rather than to gene amplification. By contrast, amplification of the *MRP* gene is probably the main mechanism underlying increased *MRP* mRNA levels in both T24/ADM-1 and T24/ADM-2 cells.

The accumulation of vincristine is often decreased in cancer cell lines overexpressing P-gp or the *MDR*1 gene, and is consistent with the findings of our previous study (Nakagawa *et al.*, 1986; Shiraishi *et al.*, 1987; Kohno *et al.*, 1988). A decreased accumulation of etoposide was observed in bladder cancer cell lines overexpressing the *MRP* gene or its product. Doxorubicin in the nuclei of T24/ADM-2 cells was almost completely removed after incubation for 120 min in the absence of drug, but this was not the case in the parental T24 cells. For the most part, decreased accumulation of etoposide or doxorubicin is observed in non-P-gp-mediated drug-resistant cell lines which overexpress MRP (Zaman *et al.*, 1973; Krishnamachary *et al.*, 1993; Scheneider *et al.*, 1994). Consistent with previous reports (Cole *et al.*, 1991; Scheneider *et al.*, 1994), MRP might be somehow involved in translocation of the anti-cancer drug from nuclei into the cytoplasm rather than outward transport from cytoplasms. Marquardt and Center (1992) have demonstrated that doxorubicin is localised at perinuclear regions when the drug is removed from culture medium for non-P-gp-mediated multidrug-resistant leukaemia cells overexpressing MRP. Further study is required to determine how MRP modulates the intracellular localisation of doxorubicin or etoposide.

In our present study, the enhanced expression of the *MRP* gene, the reduced expression of the topoisomerase II and the decreased drug accumulation were all concomitantly observed in non-P-gp-mediated drug-resistant human bladder cells. Further study will determine whether *MRP* gene overexpression is obligatorily coupled to low topoisomerase II mRNA levels, as well as to decreased drug accumulation in human bladder cancer cells.

**Acknowledgements**

This work was supported by a Grant-in-Aid for Scientific Research and Cancer Research Fund from the Ministry of Education, Science and Culture, Japan, and by grants from the Medical Research Council of Canada and the Cancer Research Society. We thank Mayumi Ono, Hiroto Izumi and Takanori Nakamura for their fruitful discussions, and also Akiko Mori and Kaoru Matsuo in our laboratory for their editorial help.

**References**

ABE T, HASEGAWA S, TANIGUCHI K, YOKOMIZO A, KUWANO T, ONO M, MORI T, HORI S, KOHNO K AND KUWANO M. (1994). Possible involvement of multidrug resistance-associated protein (MRP) gene expression in spontaneous drug resistance to vincristine, etoposide and adriamycin in human glioma cells. *Int. J. Cancer*, **58**, 860-864.

BARRAND AM, HEPPELL-PARTON CA, WRIGHT AK, RABBITTS HP AND TWENTYMAN RP. (1994). A 190-kilodalton protein overexpressed in non-P-glycoprotein-containing multidrug-resistant cells and its relationship to the MRP gene. *J. Natl Cancer Inst.*, **86**, 110-117.

BECK WT, CIRTAIN MC, DANKS MK, FELSTED RL, SAFA AR, WOLVERTON JS, SUTTLE DP AND TRENT JM. (1987). Pharmacological, molecular, and cytogenetic analysis of 'atypical' multidrug-resistant human leukemic cells. *Cancer Res.*, **47**, 5455-5460.

BRADLEY G, JURANKA PF AND LING V. (1988). Mechanism of multidrug resistance. *Biochim. Biophys. Acta*, **948**, 87-128.

BUBENIK L, BARESOVA M, VIKLICKY V, JAKOUBKOVA J, SAINEROVA H AND DONNER J. (1973). Established cell line of urinary bladder carcinoma (T24) containing tumor-specific antigen. *Int. J. Cancer*, **11**, 765-773.

COLE SPC, BHARDWAJ G, GERLACH JH, MACKIE JE, GRANT CE, ALMQUIST KC, STEWART AJ, KURZ EU, DUNCAN AM AND DEELEY RG. (1992). Overexpression of a transporter gene in a multidrug-resistant human lung cancer cell line. *Science*, **258**, 1650-1654.

GOLDSTEIN LJ, GALSKI H, FOJO A, WILLINGHAM M, LAI SL, GAZDAR A, PIRKER R, GREEN A, CRIST W AND BRODEUR GM. (1989). Expression of a multidrug resistance gene in human cancers. *J. Natl Cancer Inst.*, **81**, 116-124.

GOTTESMAN MM AND PASTAN I. (1988). The multidrug transporter, a double-edged sword. *J. Biol. Chem.*, **263**, 12163-12166.

GRANT CE, VALDIMARSSON G, HIPFNER DE, ALMQUIST KC, COLE SPC AND DEELEY RG. (1994). Overexpression of multidrug resistance-associated protein (MRP) increases resistance to natural product drugs. *Cancer Res.*, **54**, 357-361.

HARRY WH, VINCENT PL AND WHITMORE WF. (1987). An overview of intravesical therapy for superifical bladder tumors. *J. Urol.*, **138**, 1363-1368.

HYDE SC, EMSLEY P, HARTSHORN MJ, MIMMACK MM, GILEADI U, PEARCE SR, GALLAGHER MP, GILL DR, HUBBARD RE AND HIGGINS CF. (1990). Structural model of ATP-binding proteins associated with cystic fibrosis, multidrug resistance and bacterial transport. *Nature*, **346**, 362-365.

KIMIYA K, NAITO S, SOEJIMA T, SAKAMOTO N, KOTOH S, KUMAZAWA J AND TSURUO T. (1992). Establishment and characterization of doxorubicin-resistant human bladder cancer cell line, KK47/ADM. *J. Urol.*, **148**, 441-445.

KIUE A, SANO T, SUZUKI K, INADA H, OKAMURA M, KIKUCHI J, SATO S, KOHNO S AND KUWANO M. (1990). Activities of newly synthesized dihydropyridines in overcoming of vincristine resistance, calcium antagonism, and inhibition of photoaffinity labeling of P-glycoprotein in rodents. *Cancer Res.*, **50**, 310-317.

KOHNO K, KIKUCHI J, SATO S, TAKANO H, SABURI Y, ASOH K AI, KUWANO M. (1988). Vincristine-resistant human cancer KB cell line and increased expression of multidrug-resistance gene. *Jpn J. Cancer Res.*, **79**, 1238-1246.

KOHNO K, SATO S, TAKANO H, MATSUO K AND KUWANO M. (1989). The direct activation of human multidrug resistance gene (MDR1) by anticancer agents. *Biochem. Biophys. Res. Comm.*, **165**, 1415-1421.

KOHNO K, TANIMURA H, NAKAYAMA Y, MAKINO Y, WADA M, FOJO AT AND KUWANO M. (1994). Cellular control of human multidrug resistance1 (MDR1) gene expression in the absence and presence of gene amplification in human cancer cells. *J. Biol. Chem.*, **269**, 20503-20508.

KOTOH S, NAITO S, YOKONIZO A, KUMAZAWA J, ASAKUNO K, KOHNO K AND KUWANO M. (1994). Inversed expression of DNA topoisomerase I gene and collateral sensitivity to camptothecin in human cisplatin-resistant bladder cancer cells. *Cancer Res.*, **54**, 3248-3252.

KRISHNAMACHARY N AND CENTER MS. (1993). The MRP gene associated with a non-P-glycoprotein multidrug resistance encodes a 190-kDa membrane bound glycoprotein. *Cancer Res.*, **53**, 3658-3661.

KRUH GD, CHAN A, MYERS K, GAUGHAN K, MIKI T AND AARONSON ST. (1994). Expression complementary DNA library transfer establishes mrp as a multidrug resistance gene. *Cancer Res.*, **54**, 1649-1652.

MARQUARDT D. AND CENTER MS. (1992). Drug transport mechanisms in the HL60 cells isolated for resistance to adriamycin: evidence for nuclear drug accumulation and redistribution in resistant cells. *Cancer Res.*, **52**, 3157-3163.

MARSH W AND CENTER MS. (1987). Adriamycin resistance in HL60 cells and accompanying modification of a surface membrane protein contained in drug-sensitive cells. *Cancer Res.*, **47**, 5080-5086.

MATSUO K, KOHNO K, TAKANO H, SATO S, KIUE A AND KUWANO M. (1990). Reduction of drug accumulation and DNA topoisomerase II activity in acquired teniposide-resistant human cancer KB cell lines. *Cancer Res.*, **50**, 5819-5824.

MIRSKI SE, GERLACH JH AND COLE SPC. (1987). Multidrug resistance in a human small cell lung cancer cell line selected in adriamycin. *Cancer Res.*, **47**, 2594-2598.

NAKAGAWA M, AKIYAMA S, YAMAGUCHI T, SHIRAISHI N, OGATA J AND KUWANO M. (1986). Reversal of multidrug resistance by synthetic isoprenoids in the KB human cancer cell line. *Cancer Res.*, **46**, 4453-4457.

NAITO S, SAKAMOTO N, KOTOH S, GOTO K, MATSUMOTO T AND KUMAZAWA J. (1992). Correlation between the expression of P-glycoprotein and multidrug-resistant phenotype in transitional cell carcinoma of the urinary tract. *Eur. Urol.*, **22**, 158-162.

ONO M, NAKAYAMA Y, PRINCLER G, GOPAS J, KUNG H AND KUWANO M. (1992). Polyoma middle T antigen or v-src desentizes human epidermal growth factor receptor function and interference by a monensin-resistant mutation in mouse Balb 3T3 cells. *Exp. Cell Res.*, **203**, 456-465.

SCHEPER RJ, BROXTERMAN HJ, SCHEFFER GL, KAAIJK P, DALTON WS, VAN HEIJININGEN T, VAN KALTEN C, SLOVAK ML, DE VRIESE E, VAN D. VALK P, MEIJER CJ AND PINEDO HM. (1993). Overexpression of a M(r) 110,000 vesicular protein in non-P-glycoprotein-mediated multidrug resistance. *Cancer Res.*, **53**, 1475-1479.

SCHNEIDAR E, HORTON JK, YANG MCH, NAKAGAWA M AND COWAN KH. (1994). Multidrug resistance-associated protein gene overexpression and reduced drug sensitivity of topoisomerase II in a human breast carcinoma MCF7 cell line selected for etoposide resistance. *Cancer Res.*, **54**, 152-158.

SHINOHARA N, LIEBERT M, WEDEMEYER G, CHANG JHC AND GROSSMAN HB. (1993). Evaluation of multidrug resistance in human bladder cancer cell lines. *J. Urol.*, **150**, 505-509.

SHIRAISHI N, AKIYAMA S, NAKAGAWA M, KOBAYASHI M AND KUWANO M. (1987). Effect of disbenzylisoquinoline (biscoclaurine) alkaloids on multidrug resistance in KB human cancer cells. *Cancer Res.*, **47**, 2413-2416.

SLOVAK ML, HO JP, BHARDWAJ G, KURZ EU, DEELEY RG AND COLE SPC. (1993). Localization of a novel multidrug resistance-associated gene in the HT1080/DR4 and H69AR human tumour cell lines. *Cancer Res.*, **53**, 3221-3225.

STERNBERG CN, YAGODA A, SCHER HI AND WATSON RC. (1988). MVAC (methotrexate, vinblastine, doxorubicin and cisplatin) for advanced transitional cell carcinoma of the urothelium. *J. Urol.*, **139**, 461-469.

TAKANO H, KOHNO K, ONO M, UCHIDA Y AND KUWANO M. (1991). Increased phosphorylation of DNA topoisomerase II in etoposide-resistant mutants of human cancer KB cells. *Cancer Res.*, **51**, 3951-3957.

TAKANO H, KOHNO K, MATUSO K, MATSUDA T AND KUWANO M. (1992). DNA topoisomerase-targeting antitumor agents and drug resistance. *Anticancer Drugs*, **3**, 323-330.

TAYA T, KOBAYASHI T, TSUKAHARA K, UCHIBAYASHI T, NAITO K, HISAZUMI H AND KURODA K. (1977). In vitro culture of malignant tumor tissue from the human urinary tract. *Jpn J. Urol.*, **68**, 1003-1010.

ZAMAN GJ, VERSANTVOORT CH, SMIT JJ, EIJDEMS EW, DE HM, SMITH AJ, BROXTERMAN HJ, MULDER NH, DE VRIES E, BAAS F AND BORST P. (1993). Analysis of the expression of MRP, the gene for a new putative transmembrane drug transporter, in human multidrug resistant lung cancer cell lines. *Cancer Res.*, **53**, 1747-1750.